# Cyclic AMP signaling promotes regeneration of cochlear synapses after excitotoxic or noise trauma

**DOI:** 10.3389/fncel.2024.1363219

**Published:** 2024-04-17

**Authors:** Sriram Hemachandran, Ning Hu, Catherine J. Kane, Steven H. Green

**Affiliations:** Department of Biology, University of Iowa, Iowa City, IA, United States

**Keywords:** cyclic AMP-dependent protein kinase, cochlea, spiral ganglion neuron, auditory nerve, synapse, regeneration, synaptopathy, noise

## Abstract

**Introduction:**

Cochlear afferent synapses connecting inner hair cells to spiral ganglion neurons are susceptible to excitotoxic trauma on exposure to loud sound, resulting in a noise-induced cochlear synaptopathy (NICS). Here we assessed the ability of cyclic AMP-dependent protein kinase (PKA) signaling to promote cochlear synapse regeneration, inferred from its ability to promote axon regeneration in axotomized CNS neurons, another system refractory to regeneration.

**Methods:**

We mimicked NICS *in vitro* by applying a glutamate receptor agonist, kainic acid (KA) to organotypic cochlear explant cultures and experimentally manipulated cAMP signaling to determine whether PKA could promote synapse regeneration. We then delivered the cAMP phosphodiesterase inhibitor rolipram via implanted subcutaneous minipumps in noise-exposed CBA/CaJ mice to test the hypothesis that cAMP signaling could promote cochlear synapse regeneration *in vivo*.

**Results:**

We showed that the application of the cell membrane-permeable cAMP agonist 8-cpt-cAMP or the cAMP phosphodiesterase inhibitor rolipram promotes significant regeneration of synapses *in vitro* within twelve hours after their destruction by KA. This is independent of neurotrophin-3, which also promotes synapse regeneration. Moreover, of the two independent signaling effectors activated by cAMP – the cAMP Exchange Protein Activated by cAMP and the cAMP-dependent protein kinase – it is the latter that mediates synapse regeneration. Finally, we showed that systemic delivery of rolipram promotes synapse regeneration *in vivo* following NICS.

**Discussion:**

*In vitro* experiments show that cAMP signaling promotes synapse regeneration after excitotoxic destruction of cochlear synapses and does so via PKA signaling. The cAMP phosphodiesterase inhibitor rolipram promotes synapse regeneration *in vivo* in noise-exposed mice. Systemic administration of rolipram or similar compounds appears to provide a minimally invasive therapeutic approach to reversing synaptopathy post-noise.

## Introduction

The spiral ganglion neurons (SGNs) in the cochlea transmit auditory sensation from the sensory hair cells to the brain reviewed in Nayagam et al. (2011). About 95% of the SGNs synapse on inner hair cells (IHCs). Exposure to high-intensity sound can destroy these synapses as well as the cochlear hair cells themselves. However, even at sound levels too low to permanently impair hair cell function, the synapses are vulnerable. Such noise-induced loss of cochlear synapses, also termed noise-induced cochlear “synaptopathy” (NICS), is apparent when counting IHC synapses in histological preparations of cochleae from noise-exposed animals in which the hair cells survive (Kujawa and Liberman, 2009; Lin et al., 2011). The SGNs themselves survive for many weeks after loss of their peripheral synapses (Kujawa and Liberman, 2006).

NICS can be detected in live noise-exposed animals by changes in the auditory brainstem response (ABR). After noise exposure, there is a temporary elevation of auditory threshold (temporary threshold shift, TTS) assessed by ABR and, if the noise is loud enough to cause hair cell loss, there will be a permanent elevation of auditory threshold (permanent threshold shift, PTS) (Ryan et al., 2016). If there is synapse loss only and no hair cell loss, the ABR threshold generally recovers after the TTS and there is no PTS. However, the amplitude of wave-I of the ABR, the wave corresponding to spike activity in the SGNs, is permanently reduced (Kujawa and Liberman, 2009; Lin et al., 2011). Thus, a permanent reduction of ABR wave-I amplitude without PTS after noise exposure suggests NICS without hair cell loss.

Several studies have shown NICS to be due to excitotoxic damage to the synapses caused by excessive release of glutamate from IHCs exposed to high-intensity sound. Intracochlear perfusion with glutamatergic agonists causes swelling and degeneration of postsynaptic boutons on the IHCs, resembling the consequences of noise exposure (Pujol et al., 1985; Puel et al., 1998). Compromising glutamate reuptake by knockout of the glutamate transporter GLAST exacerbates NICS (Hakuba et al., 2000); conversely, synapse loss after noise exposure can be mitigated by selective blockade of glutamate receptors (Puel et al., 1994; Hu et al., 2020) or by prevention of glutamate release from IHCs by VGluT3 knockout (Kim et al., 2019). Similarly, exposure of organotypic cochlear explants cultures to the glutamate receptor agonist kainic acid (KA) *in vitro* causes synapse degeneration resembling that which occurs after noise exposure *in vivo* (Wang and Green, 2011). This provides an *in vitro* model to facilitate the assessment of compounds that can prevent synaptopathy or promote regeneration of synapses after excitotoxic trauma. Indeed, the regeneration of synapses in the cochlea is limited even though the damaged axon terminals remain immediately adjacent to the base of the IHCs (Moverman et al., 2023). It is striking that the axons are unable to grow past this small gap, and reestablish synaptic contact with the hair cells. The use of the *in vitro* model of cochlear synaptopathy showed that neurotrophic factors, BDNF or NT-3, that promote axon regeneration in other neural systems effectively promoted regeneration of IHC-SGN synapses (Wang and Green, 2011). Indeed, NT-3 has been successfully used to promote synapse regeneration after NICS *in vivo* (Wan et al., 2014; Sly et al., 2016; Suzuki et al., 2016).

Delivery of NT-3 or other peptides intracochlearly requires invasive measures. Thus, comparably effective agents that are small molecules, which can be delivered systemically and reach the cochlea through the circulation, are prime candidates for post-noise therapy. Here we use *in vitro* and *in vivo* models of cochlear synaptopathy to investigate, as such candidates, compounds that stimulate signaling by the second messenger 3′,5′-cyclic adenosine monophosphate (cAMP). Cyclic AMP is an important second messenger that, among many intracellular functions, promotes axon growth (Roisen et al., 1972; Pichichero et al., 1973) and has been shown to promote axon regeneration within the central nervous system (CNS) (Hannila and Filbin, 2008), otherwise recalcitrant to axonal regeneration. By promoting growth cone advance, cAMP, acting via cyclic AMP-dependent protein kinase (PKA), is a crucial determinant in decisions within axonal growth cones as to whether external cues are attractive or repulsive (McFarlane, 2000). Reduced PKA activity can result in cues that are typically chemoattractants, including neurotrophins, to become chemorepulsive (Wang and Zheng, 1998; Gallo et al., 2002) while increased PKA activity can prevent repulsion of axon growth by chemorepulsive cues (Song et al., 1997, 1998; Cai et al., 2001). This ability of PKA activity to overcome cues that inhibit axon growth allows agents that activate PKA to elicit axon growth on chemorepulsive surfaces such as CNS myelin (Song et al., 1998; Cai et al., 1999, 2001; Bandtlow, 2003) and is therefore of great interest with regard to promotion of axon regeneration in injured spinal cord (Qiu et al., 2002; Hannila and Filbin, 2008; Chan et al., 2014). Moreover, the ability of neurotrophic factors to promote axon regeneration has been shown to require PKA activity (Cai et al., 1999; Gallo et al., 2002) and synergize with PKA activity (Lu et al., 2004).

Here, we hypothesize that, like axonal regeneration in the CNS, the poor regeneration of synapses after NICS is due to a chemorepulsive inhibitory cue that can be overcome by activation of PKA in SGNs. We show that two cell membrane-permeable compounds that can activate PKA signaling – a cAMP agonist and an inhibitor of 3′,5′-cyclic nucleotide phosphodiesterase 4 (PDE4) expressed in the inner ear (Degerman et al., 2017; Mittal et al., 2017) – both promote synaptic regeneration *in vitro* and the latter can promote synapse regeneration after NICS *in vivo* when delivered systemically.

## Materials and methods

### Animals

For experiments using organotypic cochlear cultures, we used Sprague-Dawley rat pups from our breeding colony. Animals exposed to noise were 12–14-week-old male and female CBA/CaJ mice from our breeding colony. All of the animals were housed under a regular 12-h day/night cycle with continuous access to water and food. Noise exposures were at about the same time of day for all mice. All experimental procedures were approved by the University of Iowa Institutional Animal Care and Use Committee and were performed following the relevant guidelines and regulations of the University of Iowa Institutional Animal Care and Use Committee and is reported in accordance with ARRIVE guidelines.^1^

### Noise exposure and experimental groups

The mice were exposed to noise using a paradigm similar to that described by Kujawa and Liberman (2009): 100 dB SPL, octave-band noise, 8–16 kHz for 2 h. The noise was generated with a RZ6 multi I/O processor, a high-frequency power amplifier (IPR-1600 DSP, Peavey Electronics Corporation, Meridian, MS, United States), a high-frequency loudspeaker (Beyma driver CP21F 1 inch HF slot tweeter, Valencia, Spain) in a custom-made sound-proof chamber with the high-frequency speaker built into its top. The noise exposure was controlled using RpvdsEx software (Version 5.6, Tucker-Davis Technologies, Inc., Alachua, FL, United States).

Two mice awake and unrestrained, each in a small iron-wire cage, were positioned head-to-head below the center of the speaker. The noise level was monitored with a 1/4 inch condenser microphone (Model 7017, ACO Pacific, Inc., Belmont, CA, United States) placed at the center of the space between the two animals at the approximate level of the animals’ ears. The variation of noise levels between the animals’ ears and over the time of exposure was <1 dB.

In general, we have two criteria for exclusion of animals based on ABR (see below). (1) Temporary threshold shift <30 dB at 16 kHz, which would suggest a possibility that the noise exposure was not sufficient to cause synaptopathy. (2) Permanent threshold shift, defined as detectable threshold elevation at post-noise day 14, which would suggest that the noise damage was not limited to synaptopathy.

### Systemic administration of rolipram

One day after the noise exposure, the mice were divided into two experimental groups: a DMSO control group and a Rolipram-treated group. All mice were anesthetized during the procedure using continuous inhalation of a 0.5%–5% isoflurane/oxygen mixture. The fur was shaved from the incision site at the lower neck area and the site was cleaned and antiseptic applied. An incision ∼1 cm in length was made and a subcutaneous tunnel then made from the incision site down to the lower back. Because rolipram has been reported to be eliminated rapidly after injection (Krause and Kühne, 1988), we chose to continuously infuse rolipram from an implanted minipump. A sterilized minipump was inserted into the subcutaneous tunnel. The minipumps used in this study were Alzet type 2002 minipumps (3 × 0.7 cm in size) designed for use in small animals such as adult mice. They have a flow rate of 0.5 μl/h and a capacity to deliver contents for 2 weeks. The incision was closed using absorbable suture. A local analgesia was applied to the incision site and an iodine-containing antiseptic applied directly to the wound area after suturing the skin. After recovery, the mouse was returned to the animal facility and its condition checked daily post-surgery. The minipumps were filled with either control vehicle only (DMSO) or with rolipram (Sigma; catalog #557330) dissolved in DMSO to deliver 4.4 mg/kg/day. The mice were injected intraperitoneally with 4.4 mg/kg rolipram immediately after implantation of the minipump to elevate rolipram concentration immediately while the minipump was more gradually ramping up rolipram concentration. This specific dose for the *in vivo* studies was estimated based on the results of the *in vitro* dose response study described in section “Results” and was within the range of rolipram dosages used in previous *in vivo* studies (Siuciak et al., 2007; Kraft et al., 2013).

Reported side-effects in some patients receiving PDE inhibitors have included nausea or vomiting (Spina, 2008). However, we did not observe any such signs of distress in the mice receiving rolipram at this dosage and they showed normal weight gain. At the time of euthanasia (14–16 weeks of age), the mean (± SD) weight for rolipram-treated mice was 26.7 ± 3.3 g, not significantly different from DMSO-only control mice, 29.2 ± 7.5 g.

### Measurement of auditory brainstem response

Our procedure for measuring ABR has been described in detail previously (Hu et al., 2020) and is briefly summarized here. Responses were recorded from anesthetized mice from 90 dB SPL to 10 dB below the threshold level in 10 dB steps, and repeated in 5 dB steps near-threshold, the lowest stimulus level that evokes an identifiable ABR wave-I. The wave-I amplitude was measured between the first positive maximum to the following negative minimum. Wave-I amplitudes as a function of stimulus intensity were fitted by a two-order polynomial function for group comparisons. A RZ6 multi I/O processor with BioSigRZ software, a RA4PA 4 channel preamplifier, and a MF1 speaker (Tucker-Davis Technologies, Inc.) were used to deliver the acoustic stimuli and record the response signal. The acoustic stimuli were delivered to the external auditory meatus of the mouse from the MF1 speaker via a custom-made insertion tube. These consisted of 5 ms tone-pips presented at a rate of 21/s, alternating polarities, at frequencies of 8, 16, and 32 kHz. An active needle electrode was placed at the midline of the vertex of the skull, a reference electrode at the ipsilateral mastoid, and a ground electrode in the lower back. The acquisition time was 12 ms, at a sampling rate of 25,000/s with a band-pass filter 300–3,000 Hz, averaged over 128–1,024 sweeps.

### Synapse counts in cochlear wholemounts

After the ABR measure on PND14, mice were deeply anesthetized using an intraperitoneal injection of 80–100 mg/kg ketamine/10–12.5 mg/kg xylazine. While under surgical plane of anesthesia, the mice were exsanguinated by intracardial perfusion, first with ice-cold saline, then with 4% paraformaldehyde fixative, followed by decapitation. Processing of the tissue for synapse counts in whole-mount preparations was essentially as we have previously described (Hu et al., 2020). The temporal bones were removed and dissected quickly in ice-cold PBS. Most of the bony structure around the cochlea was carefully removed to expose the cochlea, which was fixed in 4% paraformaldehyde for 12 min, washed with ice-cold PBS, and transferred to 0.12 mM EDTA for decalcification at 4°C for 48 h. After decalcification, the cochlea was permeabilized with 1% Triton X-100 in PBS for 1 h at room temperature, washed three times with 1% Triton X-100 in PBS, and blocked in PBS with 0.1% Triton X-100, 5% horse serum, 0.1% bovine serum albumin (blocking buffer) for 60 min at room temperature.

Immunofluorescent labeling used the following primary antibodies in blocking buffer at 4°C for 24 h: myosin 6 and/or myosin 7a (Sigma), 1:400, to label the hair cells; anti-CtBP2 mouse IgG1 (BD Transduction Labs), 1:400, to label the presynaptic ribbons; and anti-PSD95 (Thermo-Scientific), 1:400, to label the postsynaptic densities (PSDs) on the SGN postsynaptic boutons. Further fixation after primary antibodies was for 2 h in 4% PFA. Fluorescent secondary antibodies used were Alexa Fluor 488-goat anti-Mouse IgG1, Alexa Fluor 546 goat anti-Mouse IgG2a, and Alexa Fluor 633 goat anti-rabbit in blocking buffer incubated at 4°C for 24 h in blocking buffer.

After immunolabeling the cochlea was cut into 5–6 pieces and mounted on a glass coverslip and imaged using a Leica SPE confocal microscope. First, a low magnification image was acquired to measure the lengths of the pieces and align them to the mouse tonotopic map according to Muller’s length-frequency equation (Muller et al., 2005). Using the Muller equation, the 8, 16, and 32 kHz locations of the cochlea were identified and imaged at higher magnification with a 63× / 1.3 NA oil objective and additional 3× electronic magnification to obtain clear images of synapses. For counting purposes, we operationally define a synapse as a colocalized presynaptic ribbon and PSD. All synapses on all hair cells wholly included in the field were counted and the synapse number divided by the number of IHCs in the field to obtain synapses/IHC. The counts were made, using ImageJ, in 3D image stacks in which the optical sections in the *x*-*y* plane (“*z*-sections”) were captured at a spacing of 0.3 μm along the *z*-axis. The representative images shown in the figures in this paper are the maximum intensity *Z*-projections of such stacks although, for counting, we used the original image stacks, not the *Z*-projections. An ImageJ macro was used to assign a random eight-digit number to all images so the person counting was unaware of the experimental group.

### Cochlear organotypic culture

Organotypic cochlear explant cultures were used for synapse counts following excitotoxic trauma *in vitro*. Our procedure for preparation of organotypic cochlear explant cultures has been described in detail previously (Wang and Green, 2011) and is briefly summarized here. After euthanasia by decapitation, the cochleae of postnatal day 5 (P5) rat pups of both sexes were removed and dissected in ice-cold PBS. The middle turn of the cochlea was used for these experiments to reduce variability due to physiological differences along the tonotopic axis and because this region corresponds to the region of the cochlea targeted by the octave-band noise exposure *in vivo*. After separating the membranous labyrinth from the modiolus, the non-sensory regions were removed using fine forceps, leaving just the organ of Corti and the corresponding part of the spiral ganglion intact. Each explant was transferred to a separate well of a multichambered coverglass system (Lab-Tek; catalog #155411) where it was positioned on a glass coverslip coated with polyornithine (100 μg/ml; Sigma; catalog #P3655) and laminin (20 μg/ml; Gibco; catalog #23017015). The explants were cultured in DMEM/N2/20% fetal bovine serum (Gibco; catalog #11965092/Gibco; catalog #17502048/Gibco; catalog #26140079) at 37°C, 6.5% CO_2_.

The explant was left undisturbed for ∼16 h to allow it to firmly attach to the coverslip. We have previously described (Wang and Green, 2011) 2 h treatment with 0.5 mM kainic acid (KA; Tocris; catalog #0222) to cause excitotoxic trauma. After the washout of the KA, the explants were maintained for up to 72 h, as indicated in the individual figures, in control culture medium or in culture medium with experimental agents added. Experimental agents used in this study were 8-cpt-cAMP (1 mM; Sigma; catalog #c3912), a stable cell membrane-permeable cAMP agonist; 8-cpt-2-O-Me-cAMP (100 μM; Sigma; catalog #c8988) a stable cell membrane-permeable cAMP agonist selective for the exchange protein activated by cAMP (EPAC) pathway; rolipram (2.4 μM; Sigma; catalog #557330), a PDE4 inhibitor; 50 ng/ml Neurotrophin-3 (NT-3, Cell Signaling; catalog #5273); 2 μg/ml TrkC-IgG, a selective NT-3 antagonist was produced in our lab (Wang and Green, 2011); H-89 (20 μM; Sigma; catalog #371963-M) a PKA inhibitor. Inhibitors, TrkC-IgG or H89, were added to the cultures 15 min prior to the addition of other experimental agents to ensure that these inhibitors were already in place.

After experimental treatment for the appropriate duration, the cultures were fixed using 4% paraformaldehyde for 7 min, then permeabilized with 1% Triton X-100. Immunolabeling of hair cells, PSDs, and presynaptic ribbons, followed by confocal imaging, randomization, and synapse counting, were done as described above for cochlear wholemount preparations.

## Results

### cAMP induces synapse regeneration *in vitro* via cAMP-dependent protein kinase signaling

**FIGURE 1:**
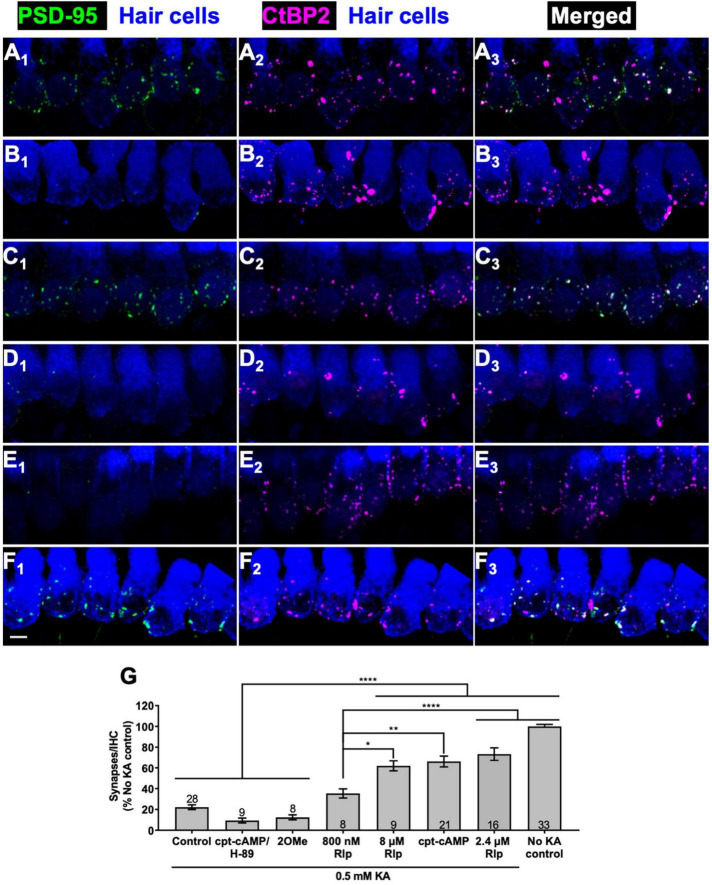
Cyclic AMP-dependent protein kinase signaling promotes synapse regeneration. **(A–F)** Examples of organotypic cochlear cultures: middle region of P5 rat cochleae exposed to 0.5 mM KA for 2 h and fixed after an additional 72 h post-KA exposed to the indicated experimental treatments: **(A)** no kainate exposure control; **(B–F)** 0.5 mM kainate; **(B)** control, no added factors; **(C)** 1 mM cpt-cAMP; **(D)** 1 mM cpt-cAMP with 20 μM H-89; **(E)** 0.5 mM 100 μM 8-cpt 2MeO cAMP; **(F)** 2.4 μM rolipram. In this figure and all following figures showing confocal images, what is shown in each image is a projection of the 3D volume to a single plane (*Z*-projection), the actual synapse counts were done on the original 3D confocal image stacks. The explants were labeled to detect postsynaptic densities (PSDs, anti-PSD95), presynaptic ribbons (anti-CtBP2), and hair cells (anti-myosin VI). Column 1 shows PSDs (green) and hair cell labeling (blue); column 2 shows ribbons (magenta) and hair cell labeling (blue); column 3 shows merged hair cell, ribbon and PSD labeling to show colocalization of PSDs and ribbons. Scale is the same for all images; scale bar, 20 μm (panel **F_1_**). **(G)** Synapses/IHC present 72 h after a 2 h exposure to KA, relative to cultures not exposed to KA (expressed as a percentage). Shown are means ± SEM for the indicated number of organotypic cochlear explant cultures. Significance of differences among conditions for each value of kainate was determined by ANOVA with Tukey’s multiple comparisons test: *p < 0.05, **p < 0.01, ****p < 0.0001. After KA, cultures were maintained in medium containing CPT-cAMP (1 mM), CPT-cAMP (1 mM) and PKA inhibitor H89 (20 μM), EPAC-selective activator 8-pCPT-2-O-Me-cAMP (100 μM), or PDE4 inhibitor rolipram (Rlp) at the three concentrations indicated.

As an *in vitro* model for NICS, we have previously shown that exposing organotypic cochlear explant cultures to the excitotoxin kainic acid results in loss of afferent synapses on IHCs (Wang and Green, 2011). The PSDs are nearly all gone by the end of a 2 h exposure to excitotoxins although presynaptic ribbons remain. We used this model system to show that neurotrophins NT-3 and BDNF promote synapse regeneration *in vitro* (Wang and Green, 2011) and here use it to ask whether cAMP signaling can promote synapse regeneration and, if so, by which signaling pathway. As described in section “Materials and methods,” the cochleae are cultured overnight, exposed to 0.5 mM KA for 2 h, cultured for a further 3 days in experimental or control media, then fixed and imaged to assess synapse regeneration. Representative images are shown in Figure 1 for control cultures and cultures maintained with modulators of cAMP signaling after excitotoxic destruction of synapses.

Figure 1A shows images of a representative control culture not exposed to KA (No KA control) that can be contrasted with comparable images of a KA-treated culture, 3 days post-KA, shown in Figure 1B. KA treatment causes a near-complete loss of PSDs (visualized by PSD95 immunoreactivity), although the presynaptic ribbons (visualized by CtBP2 immunoreactivity) persist even 3 days post-KA, as we have previously shown (Wang and Green, 2011). As shown in Figure 1C, maintaining the cultures in the cell-permeant cAMP agonist, 8-cpt-cAMP (1 mM) during the 3-day post-KA period restores a significant number of PSDs. The regenerated PSDs colocalize with the synaptic ribbons implying that these are synapses. For KA-treated cultures, the number of synapses (operationally defined as colocalized PSDs and ribbons) in cultures maintained in 8-cpt-cAMP is significantly greater than in cultures with no added 8-cpt-cAMP (Figure 1G).

Intracellular signaling initiated by cAMP utilizes at least two distinct cAMP-responsive effectors, both of which can be activated by 8-cpt-cAMP: the Exchange Protein Activated by cAMP (EPAC) (Robichaux and Cheng, 2018) and cAMP-dependent protein kinase (protein kinase A, PKA) (Taylor et al., 2013). Having implicated cAMP signaling, we asked which of these alternative effectors is utilized to effect cochlear synapse regeneration. As shown in Figures 1D, G, the EPAC-selective cAMP agonist, 8-cpt-2-O-Me-cAMP, fails to promote synapse regeneration. Maintenance of KA-exposed cochlear explants in 8-cpt-2-O-Me-cAMP results in synapse numbers significantly lower than maintenance in 8-cpt-cAMP and not significantly different from control KA-exposed cochlear explants. This implies that cAMP promotes regeneration via the PKA pathway rather than the EPAC pathway. To test that directly, we added the PKA inhibitor H-89 to cultures maintained in 8-cpt-cAMP. H-89 abrogated the effect of 8-cpt-cAMP on synapse regeneration, as can be seen by the lack of PSDs in Figure 1D and the quantified data in Figure 1G.

We next assessed the effect on synapse regeneration of an alternative means of activating PKA: raising intracellular cAMP levels by inhibiting cyclic AMP phosphodiesterase (PDE), the enzyme that metabolizes cAMP. A particular isoform of PDE, PDE type 4 (PDE4), is ubiquitously and highly expressed in nervous tissue (Bolger et al., 1993; Omori and Kotera, 2007) so is likely to play a major role in the metabolism of cAMP in neurons. PDE4 is selectively inhibited by the compound rolipram (Weishaar et al., 1985), which has been previously used as neuroprotective agent to promote SGN survival *in vitro* (Hegarty et al., 1997; Bok et al., 2003; Kranz et al., 2014; Glueckert et al., 2015).

We tested rolipram at 0.8, 2.4, and 8 μM for ability to promote synapse regeneration. Rolipram effectively promoted a significant recovery of synapses post-KA (Figure 1F). The quantified data in Figure 1G shows that 2.4 or 8 μM rolipram were approximately equally effective in promoting synapse regeneration, comparable to 1 mM 8-cpt-cAMP with synapse numbers restored to 60%—0% of the No-KA control. Rolipram at 0.8 μM was effective but significantly less so than at 2.4 μM. These results are consistent with a reported ED_50_ ≈ 0.8 μM for the particular rolipram stereoisomer that we used (Underwood et al., 1994).

### Rolipram-induced synapse regeneration is maximal within 12 h

**FIGURE 2:**
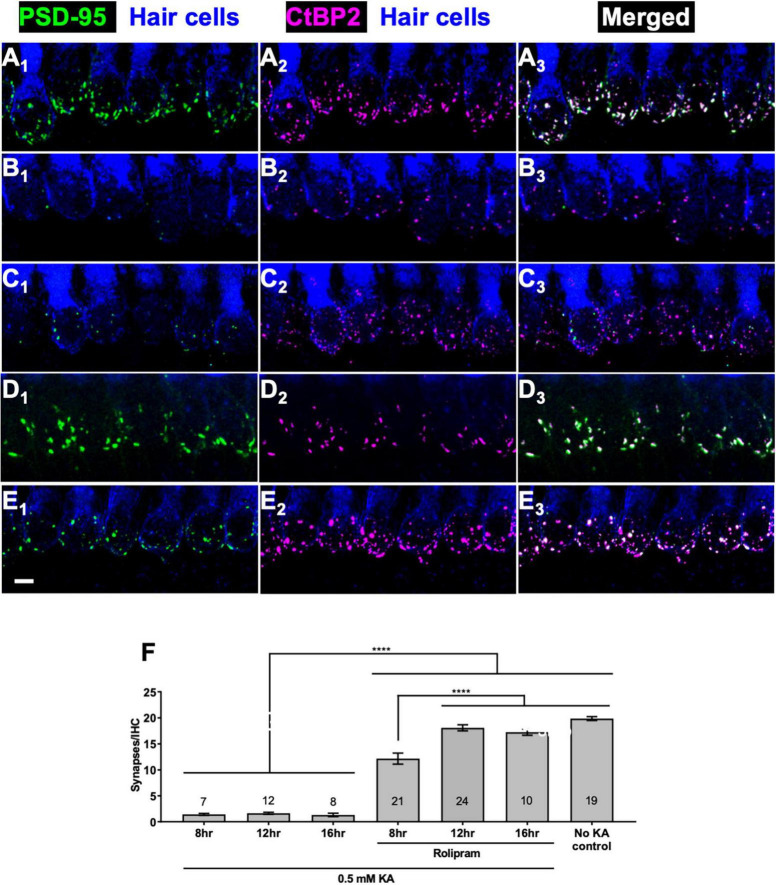
Rolipram promotes synapse regeneration within 12 h. Explants were labeled to detect SGN peripheral axons, postsynaptic densities, presynaptic ribbons, and hair cells and representative images displayed as described in Figure 1. **(A)** No KA control; **(B–E)** 2 h exposure to 0.5 mM KA followed by 16 h in control medium with no added factors **(B)** or in medium with 2.4 μM rolipram for 8 h **(C)** or 12 h **(D)** or 16 h **(E)**. All panels are at the same magnification. Scale is the same for all images; scale bar, 20 μm (panel **E_1_**). **(F)** Quantitation of synapses/IHC present 8, 12, or 16 h after a 2 h exposure to KA for cultures maintained with or without rolipram (2.4 μM). Shown are means ± SEM for the indicated number of organotypic cochlear explant cultures. The significance of differences among conditions was determined by ANOVA with Tukey’s multiple comparisons tests: ****p < 0.0001.

In the preceding experiments, a 3 day period was allowed for synapse regeneration because we had previously allowed a 3 day period for neurotrophin-induced regeneration (Wang and Green, 2011). To investigate whether cAMP-induced regeneration might occur more quickly than this, we compared cultures maintained up to 16 h post-KA in control medium to cultures maintained post-KA in medium containing 2.4 μM rolipram. We fixed the cultures 8, 12, or 16 h after washout of KA and representative images of these cultures are shown in Figures 2A–E. Cultures maintained without rolipram post-KA show no evidence of synapse regeneration (Figure 2F) but cultures maintained in rolipram show synapse regeneration within 8 h post-KA, which reaches its maximum extent by 12 h post-KA.

### Rolipram-induced cochlear synapse regeneration does not require NT-3

Our previous study of cochlear synapse regeneration (Wang and Green, 2011) showed that NT-3, the major neurotrophin expressed in the mature organ of Corti (Bailey and Green, 2014), is necessary for cochlear synapse regeneration *in vitro*. Specifically, sequestration of endogenous NT-3 using TrkC-IgG significantly diminished the ability of BDNF to promote synapse regeneration when added to the culture medium. Here we asked whether NT-3 signaling is necessary for promotion of synapse regeneration by cAMP.

**FIGURE 3:**
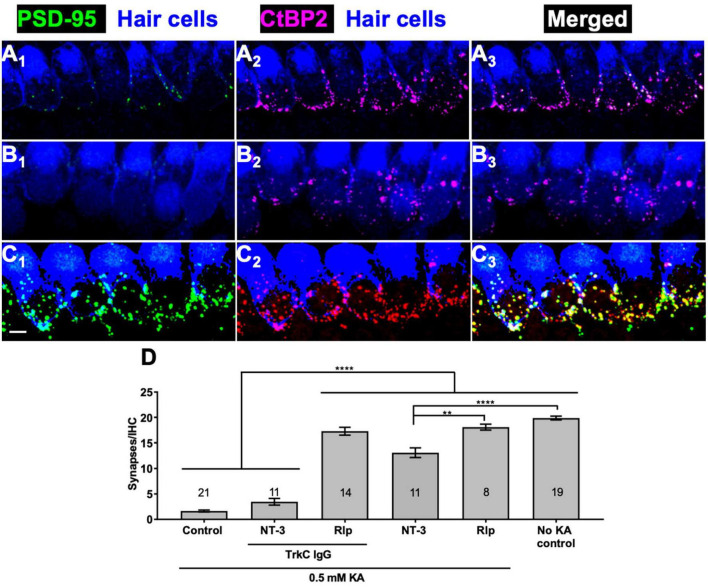
Rolipram-induced synapse regeneration does not require endogenous NT-3. TrkC-IgG was used to block TrkC signaling. Explants were labeled to detect postsynaptic densities, presynaptic ribbons, and hair cells and representative images displayed as described in Figure 1. Synapses were allowed to regenerate for 12 h after KA exposure in the indicated conditions. **(A)** NT-3 (50 ng/ml); **(B)** NT-3 (50 ng/ml), and TrkC-IgG (2 μg/ml); **(C)** rolipram (Rlp, 2.4 μM) and TrkC-IgG (2 μg/ml). All panels are at the same magnification. Scale is the same for all images; scale bar, 20 μm (lower left panel **C_1_**). **(D)** Quantification of synapses/IHC present 12 h after a 2 h exposure to KA for cultures maintained with NT-3 (50 ng/ml) or rolipram (2.4 μM) and with or without TrkC-IgG (2 μg/ml). Shown are means ± SEM for the indicated number of organotypic cochlear explant cultures. The significance of differences among conditions was determined by ANOVA with Tukey’s multiple comparisons test: **p < 0.01, ****p < 0.0001.

Sequestration of endogenous NT-3 would similarly diminish the ability of cAMP signaling to promote synapse regeneration. Possible reasons for this might include synergism between cAMP and NT-3 signaling or cAMP promoting synapse regeneration by stimulating NT-3 synthesis or secretion. We used the same strategy as in Wang and Green (2011) to block NT-3 signaling: addition of TrkC-IgG fusion protein, which binds NT-3 with high affinity and specificity, to the culture wells. The effectiveness of TrkC-IgG can be seen by comparing Figures 3A, B, which shows that synapse regeneration normally promoted by NT-3 is prevented by TrkC-IgG. In contrast, synapse regeneration promoted by rolipram is apparently not reduced by TrkC-IgG (Figures 3C, D). These data indicate that neurotrophins and cAMP use approximately equally effective, but distinct, signaling pathways to promote synapse formation.

### Rolipram promotes synapse regeneration *in vivo* following noise exposure

**FIGURE 4:**
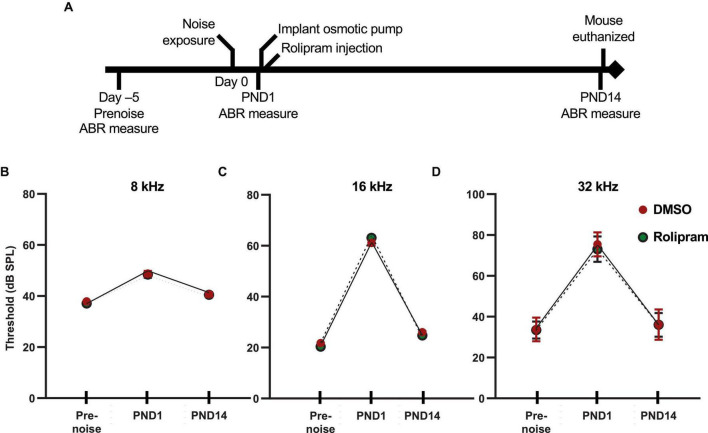
*In vivo* assessment of rolipram: timeline of ABR measures and corresponding auditory thresholds. **(A)** Timeline for the noise exposure experiments shows times relative to noise exposure (day 0) for three ABR measures: (1) prenoise, to verify normal hearing and establish a baseline for each individual mouse for normalization of post-noise measures; (2) postnoise day 1 (PND1), to measure temporary threshold shift (TTS) and verify effective noise exposure; and (3) postnoise day 14 (PND14) to ensure lack of permanent threshold shift and to measure wave-I amplitude. **(B–D)** Measures of ABR thresholds (mean ± SD) at, respectively, 8, 16, and 32 kHz for each of the three timepoints shown in panel **(A)** for noise-exposed mice implanted with subcutaneous minipumps delivering systemically only DMSO (DMSO, *n* = 23) or rolipram in DMSO (rolipram, *n* = 24). These data show a similar threshold elevation (>40 dB for 16 and 32 kHz tone pips) between prenoise and 1 day postnoise (TTS), and subsequent return to prenoise threshold by post-noise day 14. There was no significant difference in these measures between DMSO and rolipram, indicating that rolipram affects neither the TTS nor recovery from the TTS.

Having shown that rolipram can promote synapse regeneration *in vitro* following excitotoxic trauma, we next asked whether rolipram can, likewise, promote synapse regeneration *in vivo* following NICS. The design of the experiment is diagrammed in Figure 4A. The rolipram administration began 1 day after the noise exposure (post-noise day 1, PND1) to avoid any possible effects on NICS itself and to restrict this study to the effects of rolipram on post-noise synapse regeneration. The rolipram concentration was rapidly established by an injection on PND1 and then maintained continuously by an implanted minipump until PND14 when the mouse was euthanized. The rolipram in the minipump was dissolved in DMSO and the control mice were treated identically to the rolipram-treated mice except that the minipump was filled with DMSO only.

Auditory brainstem response was measured three times for each mouse (Figure 4). First, the ABR was measured 2–6 days (typically 5 days) prior to noise exposure to establish a baseline for each mouse to which post noise measures could be compared. Second, ABR was measured 1 day after noise exposure (post-noise day 1, PND1), immediately prior to the surgery to implant the minipump, to assess the effectiveness of the noise exposure. The average TTS at PND1 in our studies was 10 dB at 8 kHz, 40 dB at 16 kHz, and 50 dB at 32 kHz. Mice with a TTS <30 dB at 16 and 32 kHz were excluded from the study because of the possibility that the noise exposure was insufficient to cause synaptopathy. A final ABR measure was performed on post-noise day 14 (PND14) immediately prior to euthanasia. One purpose of the PND14 measure was to establish the lack of a PTS, i.e., that the mice used in this study had normal (same as prenoise baseline) auditory thresholds. Synaptopathy alone cannot cause significant threshold elevation (see discussion in Lin et al., 2011) so threshold elevation at PND14 implies hearing impairment due to noise damage other than synaptopathy, presumably, hair cell loss. As this study focuses exclusively on hearing impairment due to synaptopathy, ears with threshold elevation >10 dB at PND14, relative to the pre noise ABR, were excluded. The ABR measure at PND14 was also used for the quantitation of wave-I amplitude. With no PTS, a significant reduction in wave-I amplitude relative to the pre noise ABR measure implies synaptopathy. As shown in Figures 4B–D, the mice in this study showed a significant TTS at PND1 and a lack of significant PTS at PND14, relative to their pre noise ABR measures.

**FIGURE 5:**
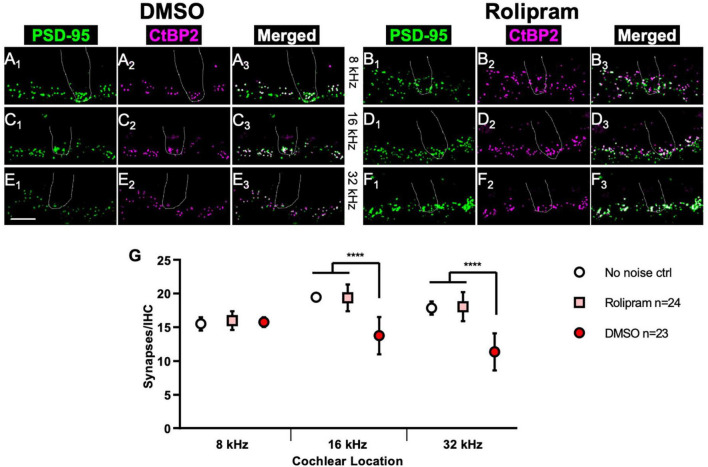
Mice treated with rolipram during noise exposure exhibit reduced synapse loss. **(A–F)** Dissected cochlear wholemount preparations were labeled (see section “Materials and methods”) to detect postsynaptic densities (green) and presynaptic ribbons (magenta), using the same antibodies as for Figure 1. Shown are representative examples at three cochlear locations, 8 kHz **(A,B)**, 16 kHz **(C,D)**, and 32 kHz **(E,F)** from mice in which either control DMSO **(A,C,E)** or DMSO with 4.4 mg/Kg/day rolipram **(B,D,F)** was delivered starting after the noise exposure. The location of a hair cell is shown by the dotted outline in each panel. Scale is the same for all images; scale bar, 10 μm (lower left panel **E_1_**). **(G)** Quantification of synapses/IHC for noise-exposed mice treated with control DMSO vehicle only and noise-exposed mice treated with rolipram in DMSO. Shown are means ± SEM for the indicated number of ears. The significance of differences among the three experimental groups was determined by ANOVA with Tukey’s multiple comparisons test: ****p < 0.0001. Synapses/IHC for control non-noise-exposed ears (No noise ctrl) is from Hu et al. (2020).

Immediately after the PND14 ABR measure, the mice were sacrificed and the cochleae dissected for histology so the synapses could be directly counted. Figure 5 shows representative images of afferent synapses labeled in the cochlea preparations from rolipram-treated and control mice at three cochlea locations: 8, 16, and 32 kHz. Noise damage to synapses is more intense at higher frequencies (Kujawa and Liberman, 2009) so, because the noise exposure was an 8–16 kHz octave band, synapse loss at the 8 kHz location was minor (Figures 5A, G) but there was significant synapse loss in control (DMSO only) mice at the 16 and 32 kHz locations (Figures 5C, E, G). In contrast, mice receiving rolipram did not exhibit significant synapse loss (Figures 5D, F, G). Because the rolipram was administered after the noise exposure, we do not infer that rolipram was protective against noise damage but, rather, that rolipram promoted synapse regeneration after noise. This is consistent with the observation of cAMP-promoted synapse regeneration following excitotoxic destruction of synapses *in vitro*.

### Rolipram partially restores ABR wave-I amplitude following noise exposure

**FIGURE 6:**
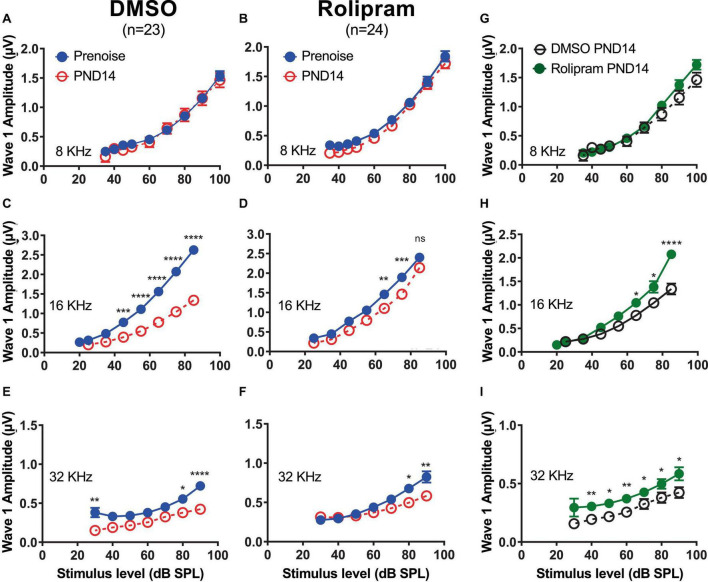
Mice treated with rolipram during noise exposure exhibit reduced decline in wave-I amplitude. **(A–F)** Measurements of ABR wave-I amplitude as a function of stimulus intensity (“growth curves”) made, on the same mice, prenoise (empty circle) and 14 days postnoise (PND14, filled circle) for 8 kHz **(A,B)**, 16 kHz **(C,D)**, and 32 kHz **(E,F)** tone pips, at indicated stimulus levels, for mice in which either control DMSO **(A,C,E)** or DMSO with rolipram **(D–F)** was delivered starting after the noise exposure. Shown are means ± SEM for the indicated number of ears. The curves were constructed by fitting the data (by least squares) to a second-order polynomial. Significance of amplitude differences between prenoise and PND14 measures at each stimulus level is as shown: *p < 0.05, **p < 0.01, ***p < 0.001, ****p < 0.0001. Repeated measure two-way ANOVA with Sidak’s multiple comparisons test for all suprathreshold stimulus levels and prenoise vs. PND14. **(G–I)** Direct comparison of PND14 wave-I amplitude between control DMSO and Rolipram groups. Significant differences between prenoise and PND14 were found at 16 and 32 kHz.

We next asked whether the restored synapses *in vivo* were functional. Because synapse loss results in a reduction in ABR wave-I amplitude, a physiological indication of functional recovery would be increased wave-I amplitude in rolipram-treated mice relative to control mice. This was tested at PND14, by which time auditory thresholds had recovered in the noise-exposed mice. The ABR was recorded at 8, 16, and 32 kHz. The ABR wave-I amplitude at PND14 for 8 kHz stimuli (Figures 6A, B) did not show a significant change from the pre noise amplitude, consistent with the lack of significant change in synapse number, nor was there any affect of rolipram (Figure 6G).

The ABR for 16 kHz tones, as expected, showed a significant decline in wave-I amplitude at PND14 in control mice relative to the pre noise measure (Figure 6C). Rolipram treatment resulted in a partial recovery of wave-I amplitude (Figure 6D) that was significant at sound intensities >60 dB SPL (Figure 6H). Similarly, at 32 kHz, the wave-I amplitude at PND14 had declined relative to the pre noise level in control mice (Figure 6E), a decline that partially recovered in rolipram-treated mice (Figures 6F, I). Rolipram appears to promote a complete or near-complete structural recovery and a partial functional recovery of cochlear synapses by PND14.

### Chronic rolipram does not itself affect the ABR or synapse numbers

**FIGURE 7:**
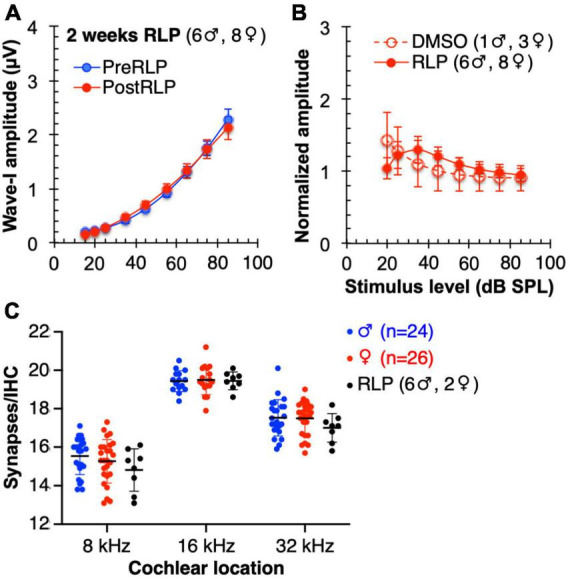
Mice chronically treated with rolipram for 2 weeks exhibit no significant changes in ABR wave-I amplitude or synapse number. **(A)** Direct comparison of wave-I amplitude measured, at 16 kHz, 1 day prior to rolipram (RLP) treatment (PreRLP) and after 2 weeks of rolipram treatment (PostRLP), assessed as summarized for Figure 6. Shown are means ± SEM. The curves were constructed by fitting the data (by least squares) to a second-order polynomial. An *F*-test was used to ask whether the pre-rolipram and 14 day rolipram data were better fit by a single curve (null hypothesis) or, alternatively, by two different curves. The conclusion (alpha = 0.05) was to accept the null hypothesis, indicating no significant difference caused by 2 weeks of rolipram exposure. **(B)** ABR wave-I amplitude was determined prior to, and after 2 weeks, of continuous rolipram treatment (RLP) or control vehicle only (DMSO). The amplitude after treatment was normalized to the amplitude prior to treatment. Shown is the mean normalized amplitude at each stimulus level for the indicated number of mice. **(C)** Quantification of synapses/IHC for male and female mice and mice treated with rolipram for 14 days (RLP). Shown are means ± SEM for the indicated number of ears at three cochlear locations, 8, 16, and 32 kHz. There was no significant difference between males and females and no significant effect of rolipram on synapse number (two-way ANOVA).

We considered the possibility that rolipram simply promotes axon sprouting in the cochlea whether or not there has been synapse loss. Such a general effect on axon sprouting could appear to be specifically rolipram-induced synapse regeneration but is rather attributable to a very different process. We therefore tested the possibility that rolipram generally induces sprouting by delivering rolipram to non-noise-exposed mice for 2 weeks using a minipump, following the identical protocol as had been used for the noise-exposed mice. Because the mice were not noise-exposed, we could include both male and female mice. As shown in Figure 7, synapse number is not significantly affected by a 2 week exposure to rolipram. Also, ABR wave-I amplitude is unaffected by this same chronic exposure to rolipram. These data imply that the apparent rescue of synapse number observed in rolipram-treated mice is not due to sprouting of undamaged axons but, rather, to repair/regeneration of damaged synapses.

## Discussion

Studies of noise-exposed mice and guinea pigs have shown that cochlear afferent synapses on the IHCs are susceptible to noise damage even at noise exposures too low to destroy the hair cells themselves (Kujawa and Liberman, 2009; Lin et al., 2011; Sergeyenko et al., 2013; Liberman, 2017). Studies of postmortem human cochleae have correspondingly shown reduced numbers of synapses on surviving hair cells (Viana et al., 2015) and the primary death of SGNs (Makary et al., 2011). In humans, this loss of synapses has been implicated in diminished speech-in-noise comprehension (Shaheen et al., 2015), and tinnitus (Schaette and McAlpine, 2011). Therefore, means to promote synapse regeneration may be valuable for reducing hearing impairment. We have shown that treatment with neurotrophic factors promotes cochlear synapse regeneration *in vitro* following synapse destruction by excitotoxic trauma (Wang and Green, 2011). This approach has been successfully extended to show synapse regeneration by NT-3 *in vivo* in mice after NICS (Wan et al., 2014; Sly et al., 2016; Suzuki et al., 2016).

Here, we take a different approach to synapse regeneration. The distal axons of the SGNs fail to regenerate their terminals and synapses, in spite of the proximity of the hair cells. We hypothesize that there might be an inhibitory signal for SGN axons analogous to inhibition of axon regeneration in the CNS. Consistent with that hypothesis, is that a function-blocking antibody to a chemorepellent axon guidance factor, RGMa, promotes synapse regeneration after noise exposure (Nevoux et al., 2021).

For axon regeneration in the CNS, the ability of neurotrophic factors to promote regeneration was found to be PKA-dependent (Cai et al., 1999). Moreover, treatment with agents that activated cAMP signaling was also capable of promoting axon regeneration (Qiu et al., 2002) via PKA (Aglah et al., 2008). While the possibility of an inhibitory signal in cochlea has not been tested directly, nevertheless, it suggested that activators of PKA might promote regeneration of cochlear synapses. Indeed, our experiments with cultured organotypic cochlear explants showed that the ability of 8-cpt-cAMP, a cell membrane-permeable cAMP mimetic, to promote synapse regeneration likewise requires PKA activity. A possible advantage over neurotrophic factors is that activators of cAMP signaling can be administered orally or systemically by injection, which would make them more amenable to therapeutic use (Chan et al., 2014). One such activator of PKA signaling is the PDE4 inhibitor rolipram, which can promote axon regeneration in the CNS (Nikulina et al., 2004; Hannila and Filbin, 2008). We showed here that rolipram is also effective in promoting cochlear synapse regeneration *in vitro*. Therefore, we assessed the ability of rolipram to promote regeneration of cochlear synapses *in vivo*.

Rolipram is a drug capable of being rapidly absorbed orally and can cross the blood-brain barrier (Krause and Kühne, 1988; Krause et al., 1990) making it a viable candidate for systemic, as opposed to intracochlear, administration. In this study, we used implanted osmotic pumps to continuously deliver the rolipram, providing a constant concentration in the mice. When assessed by PND14, the rolipram-treated mice showed a complete or near-complete restoration of synapses in those cochlear regions in which synapses had been lost in the control mice. Because delivery of rolipram was initiated a day after noise exposure, by which time synapses have degenerated (Liberman et al., 2015), the implication is that rolipram did not prevent the degeneration but promoted regeneration of synapses that had already degenerated. Nevertheless, ABR wave-I amplitude recovered significantly but not to pre-noise levels. Further investigation will be required to determine the reason(s) for this.

The second messenger cAMP has many functions in intracellular signaling and, for example, has been implicated in axonal growth through its effect on microtubule assembly (Roisen et al., 1972). Nevertheless, our studies of cultured SGNs (Hegarty et al., 1997) have shown that cAMP signaling does not promote neurite growth but, rather, inhibits neurite growth promoted by neurotrophic factors. We have also shown that synaptically targeted PKA can limit synapse formation in the brain (Lu et al., 2011). Therefore, we do not necessarily infer from the experiments summarized above that PKA signaling directly promotes synapse regeneration. However, cyclic AMP signaling has been shown to prevent or reverse the effect of chemorepellent substrate factors, facilitating chemoattraction (Song et al., 1998; Hannila and Filbin, 2008). Thus, we can suggest an alternative hypothesis that molecules intrinsic to the cochlea prevent synapse regeneration but cAMP, signaling via PKA, counteracts this inhibition. Our observations and those of Nevoux et al. (2021) are consistent with, and support such a hypothesis, but do not identify a specific inhibitory signal or rule out all alternative hypotheses. Regardless of the mechanism by which this is accomplished, rolipram does appear to be effective in promoting recovery from noise-induced cochlear synaptopathy.

Restoration of cochlear synapses by PDE4 inhibitors such as rolipram has potential advantages over the restoration of cochlear synapses by agents, such as neurotrophic factors, that must be delivered directly to the cochlea or, transtympanically, to the round window (Suzuki et al., 2016). These latter routes of delivery are more invasive and do not appear to have been effective in all subjects (Suzuki et al., 2016). Moreover, high levels of NT-3 in the cochlea may damage otherwise healthy elements (Lee et al., 2016). Rolipram and related compounds have already been suggested as possible therapeutics because of their ability to be administered systemically or even orally and stimulate cAMP signaling (Nicholson et al., 1991). They have been used, for example, to treat depression (Wachtel, 1983; Horowski, 1985) and the related PDE4 inhibitor roflumilast has FDA approval for treatment of symptoms of chronic obstructive pulmonary disorder by oral delivery (Wedzicha et al., 2016). A possible concern is that rolipram has been reported to induce endolymphatic hydrops (Degerman et al., 2017, 2019). However, this occurred when rolipram was administered in combination with vasopressin, and with a 4-week exposure time. In our experiments, synapse recovery occurred within 2 weeks of treatment with rolipram alone. Two weeks of exposure to rolipram had no effect on hearing as assessed by ABR (Figure 7). The similar compound roflumilast has been used as a therapeutic with endolymphatic hydrops not reported as an adverse reaction (Wedzicha et al., 2016).

## Conclusion

In summary, we show here that agents, including the cAMP phosphodiesterase inhibitor rolipram, that stimulate cAMP signaling promote cochlear synapse regeneration in an *in vitro* model of cochlear synaptopathy, doing so via cAMP-dependent protein kinase signaling. We further show that rolipram, delivered systemically by injection, is effective in restoring cochlear synapses *in vivo* in noise-exposed mice, without adverse effect on hearing. These results suggest that small-molecule activators of cAMP signaling, which can be delivered systemically, can have therapeutic value in the treatment of NICS.

## Data availability statement

The raw data supporting the conclusions of this article will be made available by the authors, without undue reservation.

## Ethics statement

The animal study was approved by the University of Iowa Institutional Animal Care and Use Committee. The study was conducted in accordance with the local legislation and institutional requirements.

## Author contributions

SH: Conceptualization, Data curation, Formal analysis, Investigation, Methodology, Writing – original draft, Writing – review & editing. NH: Conceptualization, Data curation, Formal analysis, Investigation, Methodology, Project administration, Writing – review & editing. CK: Data curation, Formal analysis, Methodology, Writing – review & editing. SHG: Conceptualization, Data curation, Formal analysis, Funding acquisition, Investigation, Project administration, Supervision, Writing – original draft, Writing – review & editing.

## Footnotes

1
https://arriveguidelines.org
